# Annual Circulars of Medical Colleges

**Published:** 1845-10

**Authors:** 


### ANNUAL CIRCULARS OF MEDICAL COLLEGES.

In addition to those heretofore acknowledged, we have received the following:

1.—*School of Medicine and Surgery of Montreal* (*Canada.*)—According to the circular before us, "The third sessional year of the School of Medicine and Surgery of Montreal, will commence on the first Monday in November, from which day to the 30th of April—a period of six months—*a series of Lectures*—one hundred and twenty in number—will be delivered on the following branches of Medical Science, viz:—

| | |
|---|---|
| General and Special Pathology, . . . | Dr. Badgley. |
| Midwifery, and Diseases of Women and Children, | Dr. Arnoldi, Jr. |
| Anatomy and Physiology, . . . . . | Dr. H. Nelson. |
| Surgery . . . . . . . . . | Dr. Munro. |
| Chemistry and Pharmacy, . . . . . | Dr. Sutherland. |
| Materia Medica and Therapeutics, . . . . | Dr. Bibaud. |
| Practical Anatomy, . . . . . . | Dr. Regnier. |

The Lectures will be in the English and French languages; *delivered at separate hours and in different rooms.*"

2.—*Annual Announcement of Jefferson Medical College*—1845.—The *Announcement* of this Institution for the present year, mentions no changes or modifications. The Faculty, Courses of Instruction, &c. continue as heretofore.

The number of graduates at the last commencement was 116; the number of students composing the class of the last session, was 409—sixty eight more than that of the preceding session—being the largest class of medical students ever assembled in any medical school in the United States, except the University of Pennsylvania. The most important feature in this announcement is the great number of patients that were prescribed for, and operated on, in the presence of the class, at the College Clinic. It would seem as if most of the operations known to surgery had been performed, and many of them repeatedly.

3.—*Catalogue and Circular of the Albany Medical College*—1845. We notice no change in the Faculty, courses of instruction, or general arrangements of this institution. The most interesting item of intelligence which we have noticed in this circular, is the following: "The College has received from the State, within the last four years, the sum of $16,000, all of which has been expended in improving the Building, and the purchase of Anatomical Preparations, Chemical Apparatus and Books."

When will the Medical Colleges of Pennsylvania be enabled to lift up their hands in grateful acknowledgment of such munificence? Not only all the Medical Colleges of New York, but those of nearly all the states of the Union, are liberally aided by contributions of money, apparatus, and buildings. In Pennsylvania, it is otherwise:—they are left to take care of themselves.

4.—*Annual Circular of the Washington University of Baltimore: Session*, 1845–6.—In the organization of this school, considerable changes have occurred. Dr. Annan has been transferred from the chair of Materia Medica to a chair newly created, entitled, "General Pathology, and Special Pathology, Physical Diagnosis and Treatment of Diseases of the Chest." The chair formerly occupied by Dr. Annan is filled by the appointment of Dr. Wilson; the chair of Obstetrics and Diseases of Women and Children, by the appointment of Dr. Fonerden, vice Dr. Jennings, resigned on account of "ill health and infirmities." The other chairs, we believe, are filled as heretofore.

5.—*Report on the Medical Department of the University of Pennsylvania, for the year* 1845.—This *Report* is addressed by the Faculty to the Alumni of the institution, and is intended to be issued annually hereafter. The following are the reasons assigned for this departure from the ancient practice of our Alma Mater:—"The Medical Faculty of the University of Pennsylvania offer the following statement in relation to the present condition of the school under their charge, in compliance with a wish frequently expressed on the part of their former pupils, and in accordance with their sense of what is due to the institution and its friends. A medical school, which has been in existence eighty years, which exhibits a list of graduates amounting to nearly four thousand four hundred, and has contributed to the education of eleven or twelve thousand physicians, presents a relation to the profession and to the community which is not without interest, and justifies, if it does not call for an occasional exposition of its concerns."

"The present Circular contains catalogues of the matriculants and graduates of the last session, with an account of the present officers of the school, and a brief recapitulation of its condition in relation to the number of pupils who have attended upon its instructions, and received its honors since 1834." From a comparison of its present condition with that of former years, the institution has lost none of its former prosperity; indeed, the average of attendance on the lectures, "during the last ten years, has been greater than the general average since 1810." The number of the class of the last session is 447; and of the graduates of the last year, 160.

The Clinic held at the University the two past years, at which a large number of patients was prescribed for, and numerous operations performed in the presence of the class, is to be continued, and enlarged in its operations.

6.—*Third Annual Announcement and Catalogue of the Rush Medical College, Chicago, Ill.*—No alterations in the arrangement of the faculty are noticed, or in the courses of instruction. Additions to the means of illustration in the department of chemistry are mentioned, and among the apparatus, *a fine microscope!* A Surgical Clinic was held during the last session, at which it appears that twelve operations were performed. It is to be continued.

